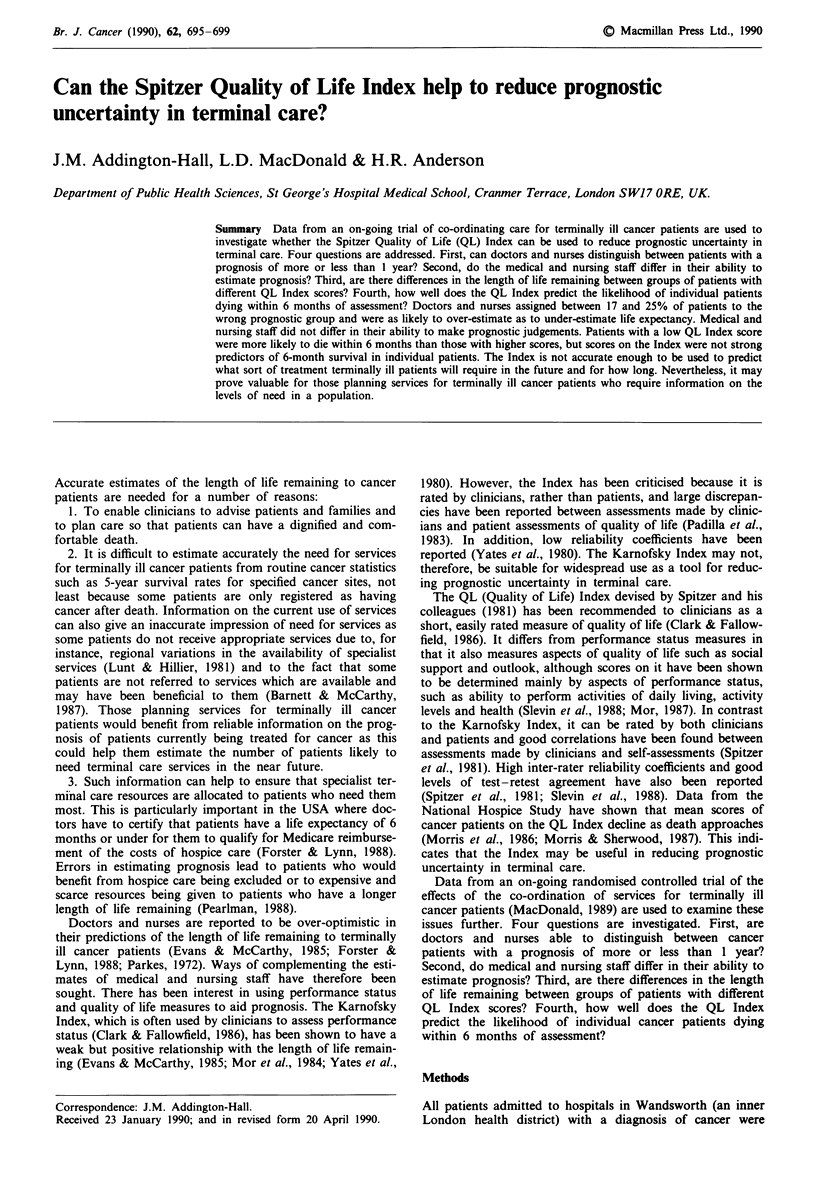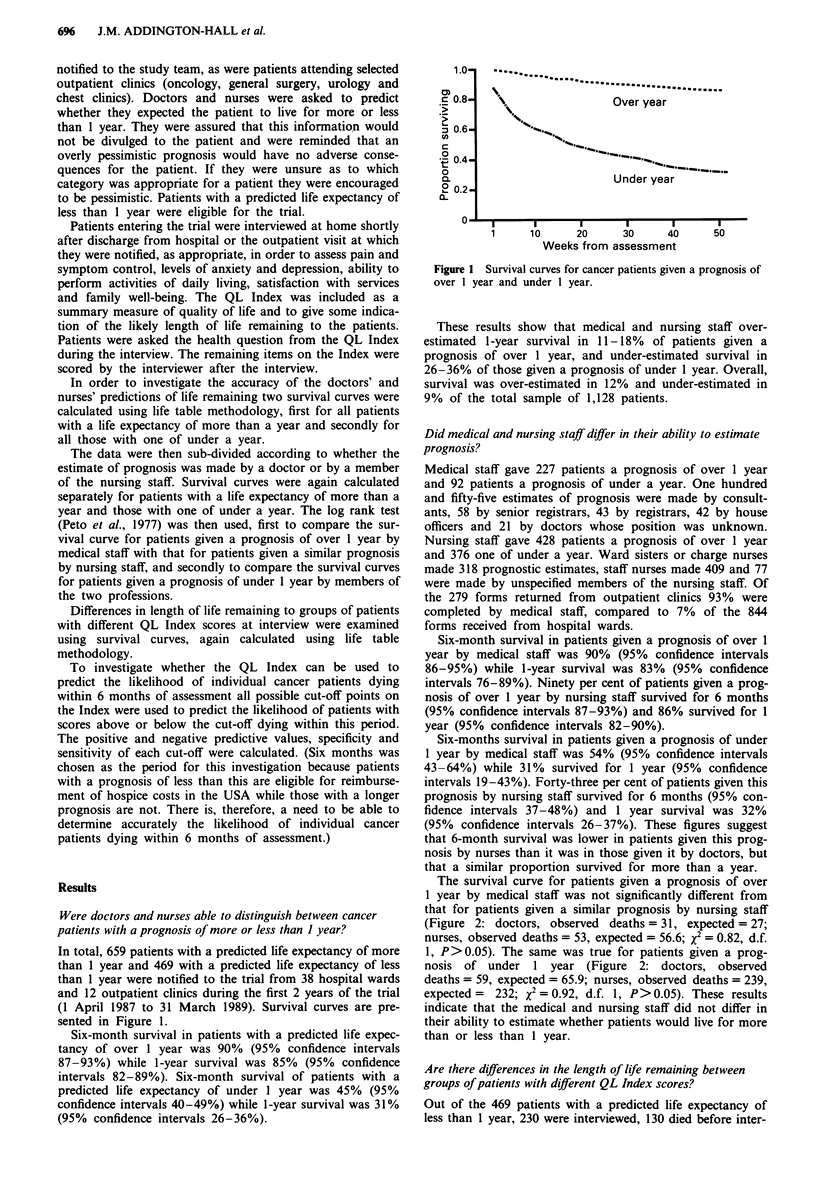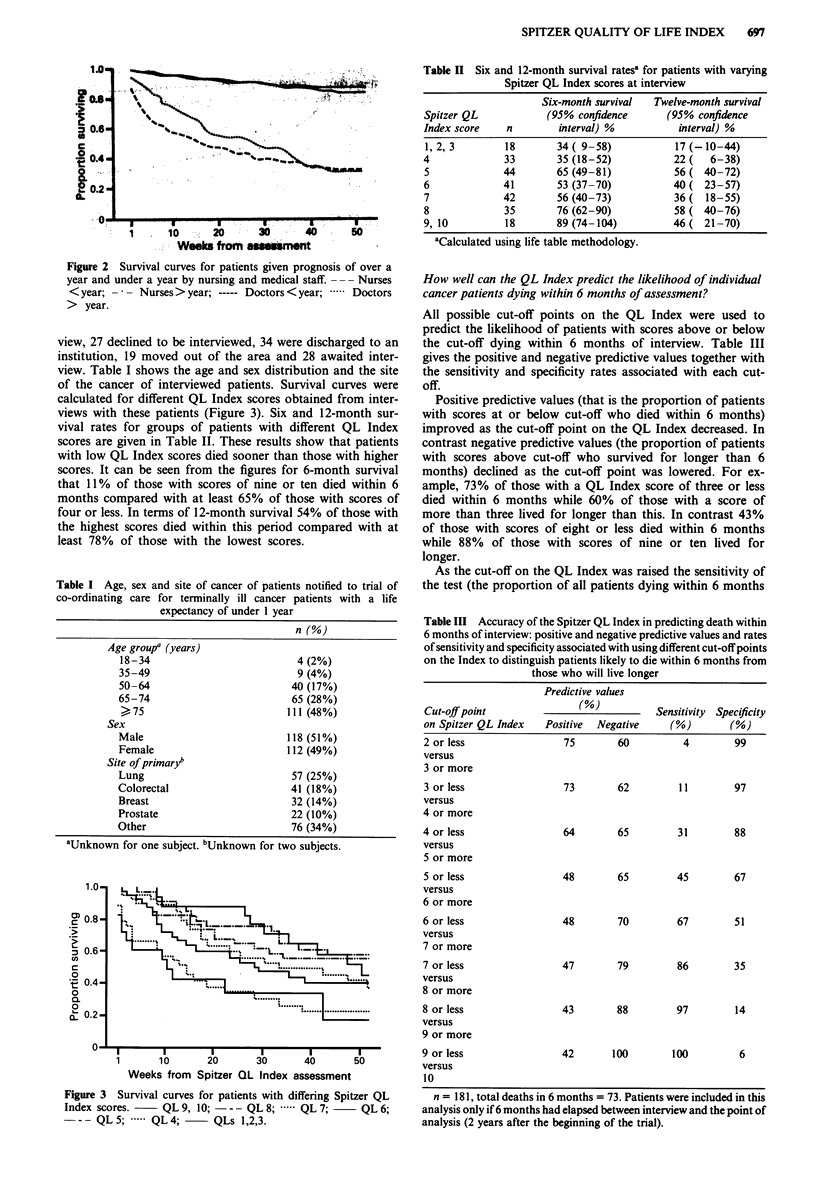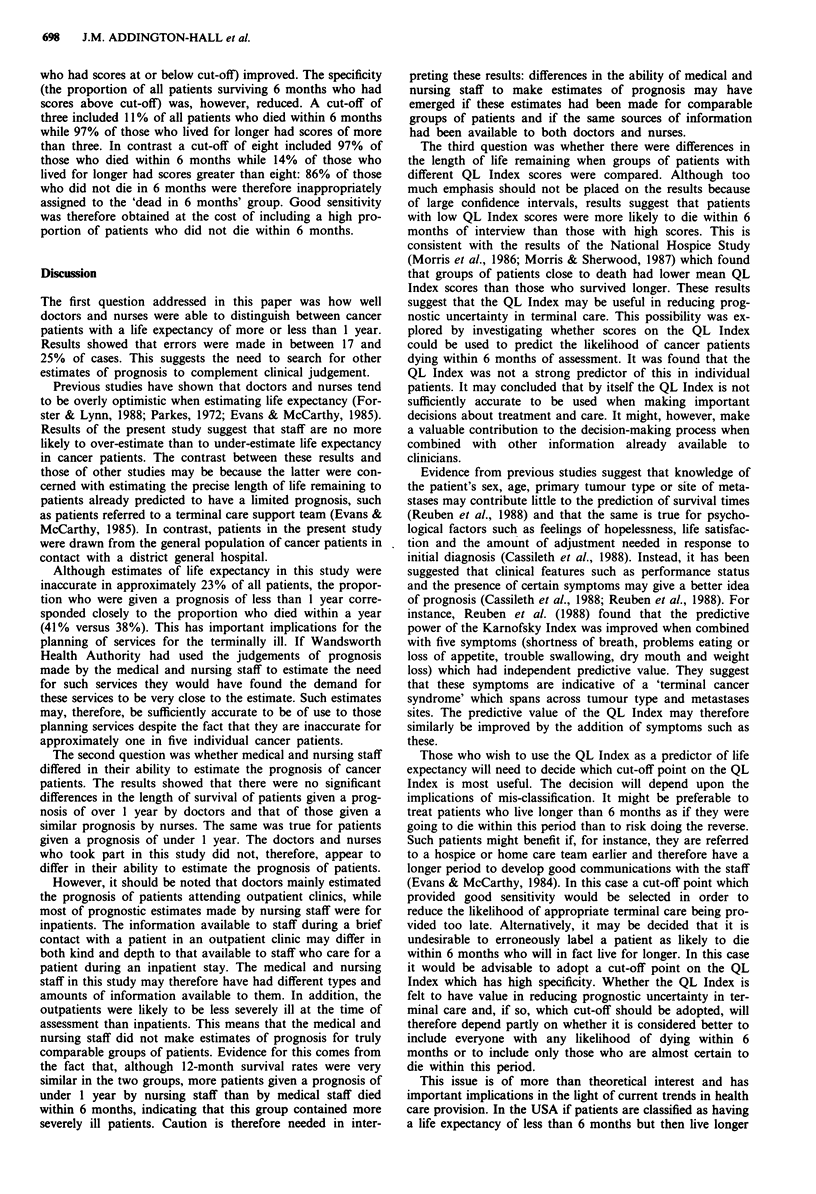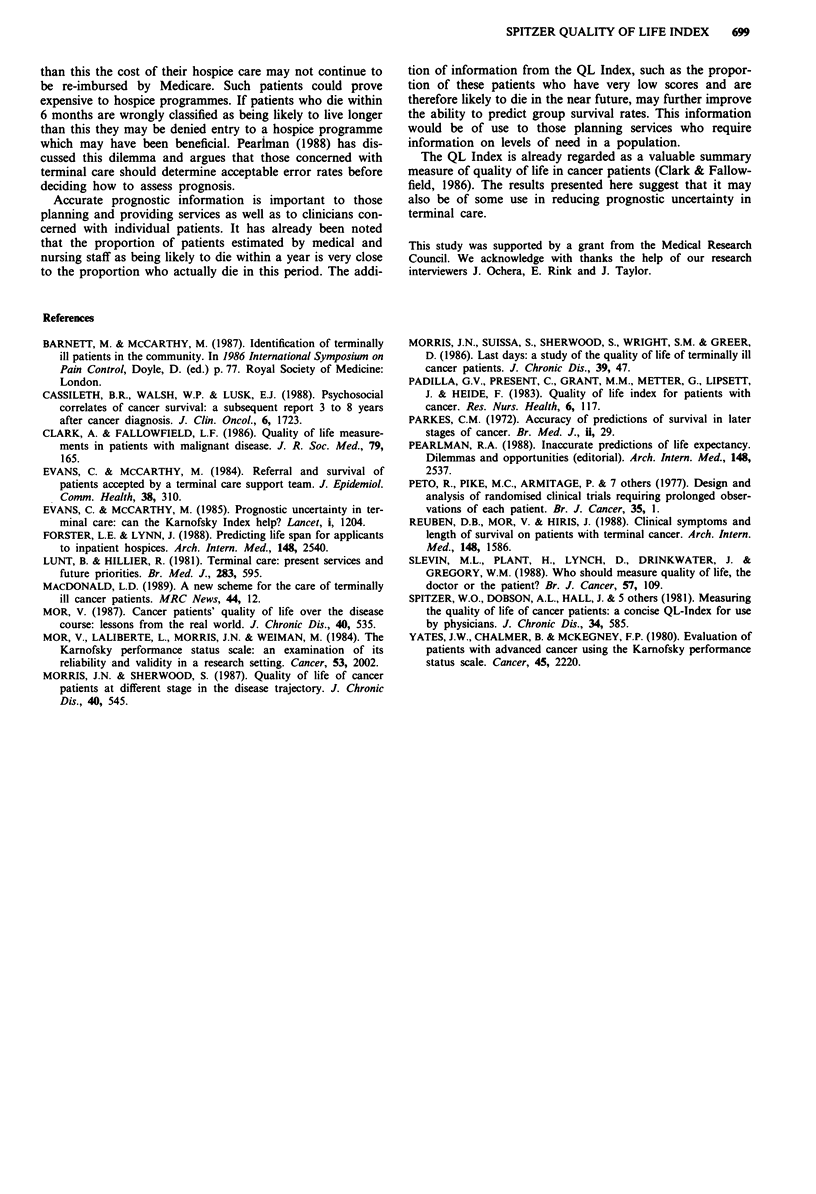# Can the Spitzer Quality of Life Index help to reduce prognostic uncertainty in terminal care?

**DOI:** 10.1038/bjc.1990.360

**Published:** 1990-10

**Authors:** J. M. Addington-Hall, L. D. MacDonald, H. R. Anderson

**Affiliations:** Department of Public Health Sciences, St George's Hospital Medical School, London, UK.

## Abstract

Data from an on-going trial of co-ordinating care for terminally ill cancer patients are used to investigate whether the Spitzer Quality of Life (QL) Index can be used to reduce prognostic uncertainty in terminal care. Four questions are addressed. First, can doctors and nurses distinguish between patients with a prognosis of more or less than 1 year? Second, do the medical and nursing staff differ in their ability to estimate prognosis? Third, are there differences in the length of life remaining between groups of patients with different QL Index scores? Fourth, how well does the QL Index predict the likelihood of individual patients dying within 6 months of assessment? Doctors and nurses assigned between 17 and 25% of patients to the wrong prognostic group and were as likely to over-estimate as to under-estimate life expectancy. Medical and nursing staff did not differ in their ability to make prognostic judgements. Patients with a low QL Index score were more likely to die within 6 months than those with higher scores, but scores on the Index were not strong predictors of 6-month survival in individual patients. The Index is not accurate enough to be used to predict what sort of treatment terminally ill patients will require in the future and for how long. Nevertheless, it may prove valuable for those planning services for terminally ill cancer patients who require information on the levels of need in a population.


Br. J. Cancer (1990), 62, 695–699 © Macmillan Press Ltd., 1990

### Can the Spitzer Quality of Life Index help to reduce prognostic uncertainty in terminal care?

J.M. Addington-Hall, L.D. MacDonald & H.R. Anderson

*Department of Public Health Sciences, St George's Hospital Medical School, Cranmer Terrace, London SW17 0RE, UK.*

**Summary** Data from an on-going trial of co-ordinating care for terminally ill cancer patients are used to investigate whether the Spitzer Quality of Life (QL) Index can be used to reduce prognostic uncertainty in terminal care. Four questions are addressed. First, can doctors and nurses distinguish between patients with a prognosis of more or less than 1 year? Second, do the medical and nursing staff differ in their ability to estimate prognosis? Third, are there differences in the length of life remaining between groups of patients with different QL Index scores? Fourth, how well does the QL Index predict the likelihood of individual patients dying within 6 months of assessment? Doctors and nurses assigned between 17 and 25% of patients to the wrong prognostic group and were as likely to over-estimate as to under-estimate life expectancy. Medical and nursing staff did not differ in their ability to make prognostic judgements. Patients with a low QL Index score were more likely to die within 6 months than those with higher scores, but scores on the Index were not strong predictors of 6-month survival in individual patients. The Index is not accurate enough to be used to predict what sort of treatment terminally ill patients will require in the future and for how long. Nevertheless, it may prove valuable for those planning services for terminally ill cancer patients who require information on the levels of need in a population.

Accurate estimates of the length of life remaining to cancer patients are needed for a number of reasons:

1. To enable clinicians to advise patients and families and to plan care so that patients can have a dignified and comfortable death.
2. It is difficult to estimate accurately the need for services for terminally ill cancer patients from routine cancer statistics such as 5-year survival rates for specified cancer sites, not least because some patients are only registered as having cancer after death. Information on the current use of services can also give an inaccurate impression of need for services as some patients do not receive appropriate services due to, for instance, regional variations in the availability of specialist services (Lunt & Hillier, 1981) and to the fact that some patients are not referred to services which are available and may have been beneficial to them (Barnett & McCarthy, 1987). Those planning services for terminally ill cancer patients would benefit from reliable information on the prognosis of patients currently being treated for cancer as this could help them estimate the number of patients likely to need terminal care services in the near future.
3. Such information can help to ensure that specialist terminal care resources are allocated to patients who need them most. This is particularly important in the USA where doctors have to certify that patients have a life expectancy of 6 months or under for them to qualify for Medicare reimbursement of the costs of hospice care (Forster & Lynn, 1988). Errors in estimating prognosis lead to patients who would benefit from hospice care being excluded or to expensive and scarce resources being given to patients who have a longer length of life remaining (Pearlman, 1988).

Doctors and nurses are reported to be over-optimistic in their predictions of the length of life remaining to terminally ill cancer patients (Evans & McCarthy, 1985; Forster & Lynn, 1988; Parkes, 1972). Ways of complementing the estimates of medical and nursing staff have therefore been sought. There has been interest in using performance status and quality of life measures to aid prognosis. The Karnofsky Index, which is often used by clinicians to assess performance status (Clark & Fallowfield, 1986), has been shown to have a weak but positive relationship with the length of life remaining (Evans & McCarthy, 1985; Mor *et al.*, 1984; Yates *et al.*, 1980). However, the Index has been criticised because it is rated by clinicians, rather than patients, and large discrepancies have been reported between assessments made by clinicians and patient assessments of quality of life (Padilla *et al.*, 1983). In addition, low reliability coefficients have been reported (Yates *et al.*, 1980). The Karnofsky Index may not, therefore, be suitable for widespread use as a tool for reducing prognostic uncertainty in terminal care.

The QL (Quality of Life) Index devised by Spitzer and his colleagues (1981) has been recommended to clinicians as a short, easily rated measure of quality of life (Clark & Fallowfield, 1986). It differs from performance status measures in that it also measures aspects of quality of life such as social support and outlook, although scores on it have been shown to be determined mainly by aspects of performance status, such as ability to perform activities of daily living, activity levels and health (Slevin *et al.*, 1988; Mor, 1987). In contrast to the Karnofsky Index, it can be rated by both clinicians and patients and good correlations have been found between assessments made by clinicians and self-assessments (Spitzer *et al.*, 1981). High inter-rater reliability coefficients and good levels of test–retest agreement have also been reported (Spitzer *et al.*, 1981; Slevin *et al.*, 1988). Data from the National Hospice Study have shown that mean scores of cancer patients on the QL Index decline as death approaches (Morris *et al.*, 1986; Morris & Sherwood, 1987). This indicates that the Index may be useful in reducing prognostic uncertainty in terminal care.

Data from an on-going randomised controlled trial of the effects of the co-ordination of services for terminally ill cancer patients (MacDonald, 1989) are used to examine these issues further. Four questions are investigated. First, are doctors and nurses able to distinguish between cancer patients with a prognosis of more or less than 1 year? Second, do medical and nursing staff differ in their ability to estimate prognosis? Third, are there differences in the length of life remaining between groups of patients with different QL Index scores? Fourth, how well does the QL Index predict the likelihood of individual cancer patients dying within 6 months of assessment?

### Methods

All patients admitted to hospitals in Wandsworth (an inner London health district) with a diagnosis of cancer were

Correspondence: J.M. Addington-Hall.
Received 23 January 1990; and in revised form 20 April 1990.

notified to the study team, as were patients attending selected outpatient clinics (oncology, general surgery, urology and chest clinics). Doctors and nurses were asked to predict whether they expected the patient to live for more or less than 1 year. They were assured that this information would not be divulged to the patient and were reminded that an overly pessimistic prognosis would have no adverse consequences for the patient. If they were unsure as to which category was appropriate for a patient they were encouraged to be pessimistic. Patients with a predicted life expectancy of less than 1 year were eligible for the trial.

Patients entering the trial were interviewed at home shortly after discharge from hospital or the outpatient visit at which they were notified, as appropriate, in order to assess pain and symptom control, levels of anxiety and depression, ability to perform activities of daily living, satisfaction with services and family well-being. The QL Index was included as a summary measure of quality of life and to give some indication of the likely length of life remaining to the patients. Patients were asked the health question from the QL Index during the interview. The remaining items on the Index were scored by the interviewer after the interview.

In order to investigate the accuracy of the doctors' and nurses' predictions of life remaining two survival curves were calculated using life table methodology, first for all patients with a life expectancy of more than a year and secondly for all those with one of under a year.

The data were then sub-divided according to whether the estimate of prognosis was made by a doctor or by a member of the nursing staff. Survival curves were again calculated separately for patients with a life expectancy of more than a year and those with one of under a year. The log rank test (Peto *et al.*, 1977) was then used, first to compare the survival curve for patients given a prognosis of over 1 year by medical staff with that for patients given a similar prognosis by nursing staff, and secondly to compare the survival curves for patients given a prognosis of under 1 year by members of the two professions.

Differences in length of life remaining to groups of patients with different QL Index scores at interview were examined using survival curves, again calculated using life table methodology.

To investigate whether the QL Index can be used to predict the likelihood of individual cancer patients dying within 6 months of assessment all possible cut-off points on the Index were used to predict the likelihood of patients with scores above or below the cut-off dying within this period. The positive and negative predictive values, specificity and sensitivity of each cut-off were calculated. (Six months was chosen as the period for this investigation because patients with a prognosis of less than this are eligible for reimbursement of hospice costs in the USA while those with a longer prognosis are not. There is, therefore, a need to be able to determine accurately the likelihood of individual cancer patients dying within 6 months of assessment.)

### Results

### *Were doctors and nurses able to distinguish between cancer patients with a prognosis of more or less than 1 year?*

In total, 659 patients with a predicted life expectancy of more than 1 year and 469 with a predicted life expectancy of less than 1 year were notified to the trial from 38 hospital wards and 12 outpatient clinics during the first 2 years of the trial (1 April 1987 to 31 March 1989). Survival curves are presented in Figure 1.

Six-month survival in patients with a predicted life expectancy of over 1 year was 90% (95% confidence intervals 87–93%) while 1-year survival was 85% (95% confidence intervals 82–89%). Six-month survival of patients with a predicted life expectancy of under 1 year was 45% (95% confidence intervals 40–49%) while 1-year survival was 31% (95% confidence intervals 26–36%).

**Figure 1** Survival curves for cancer patients given a prognosis of over 1 year and under 1 year.

These results show that medical and nursing staff over-estimated 1-year survival in 11–18% of patients given a prognosis of over 1 year, and under-estimated survival in 26–36% of those given a prognosis of under 1 year. Overall, survival was over-estimated in 12% and under-estimated in 9% of the total sample of 1,128 patients.

### *Did medical and nursing staff differ in their ability to estimate prognosis?*

Medical staff gave 227 patients a prognosis of over 1 year and 92 patients a prognosis of under a year. One hundred and fifty-five estimates of prognosis were made by consultants, 58 by senior registrars, 43 by registrars, 42 by house officers and 21 by doctors whose position was unknown. Nursing staff gave 428 patients a prognosis of over 1 year and 376 one of under a year. Ward sisters or charge nurses made 318 prognostic estimates, staff nurses made 409 and 77 were made by unspecified members of the nursing staff. Of the 279 forms returned from outpatient clinics 93% were completed by medical staff, compared to 7% of the 844 forms received from hospital wards.

Six-month survival in patients given a prognosis of over 1 year by medical staff was 90% (95% confidence intervals 86–95%) while 1-year survival was 83% (95% confidence intervals 76–89%). Ninety per cent of patients given a prognosis of over 1 year by nursing staff survived for 6 months (95% confidence intervals 87–93%) and 86% survived for 1 year (95% confidence intervals 82–90%).

Six-months survival in patients given a prognosis of under 1 year by medical staff was 54% (95% confidence intervals 43–64%) while 31% survived for 1 year (95% confidence intervals 19–43%). Forty-three per cent of patients given this prognosis by nursing staff survived for 6 months (95% confidence intervals 37–48%) and 1 year survival was 32% (95% confidence intervals 26–37%). These figures suggest that 6-month survival was lower in patients given this prognosis by nurses than it was in those given it by doctors, but that a similar proportion survived for more than a year.

The survival curve for patients given a prognosis of over 1 year by medical staff was not significantly different from that for patients given a similar prognosis by nursing staff (Figure 2: doctors, observed deaths = 31, expected = 27; nurses, observed deaths = 53, expected = 56.6; $\chi^2 = 0.82$, d.f. 1, $P > 0.05$). The same was true for patients given a prognosis of under 1 year (Figure 2: doctors, observed deaths = 59, expected = 65.9; nurses, observed deaths = 239, expected = 232; $\chi^2 = 0.92$, d.f. 1, $P > 0.05$). These results indicate that the medical and nursing staff did not differ in their ability to estimate whether patients would live for more than or less than 1 year.

### *Are there differences in the length of life remaining between groups of patients with different QL Index scores?*

Out of the 469 patients with a predicted life expectancy of less than 1 year, 230 were interviewed, 130 died before inter-

**Figure 2** Survival curves for patients given prognosis of over a year and under a year by nursing and medical staff. --- Nurses <year; -·- Nurses>year; ----- Doctors<year; ····· Doctors > year.

view, 27 declined to be interviewed, 34 were discharged to an institution, 19 moved out of the area and 28 awaited interview. Table I shows the age and sex distribution and the site of the cancer of interviewed patients. Survival curves were calculated for different QL Index scores obtained from interviews with these patients (Figure 3). Six and 12-month survival rates for groups of patients with different QL Index scores are given in Table II. These results show that patients with low QL Index scores died sooner than those with higher scores. It can be seen from the figures for 6-month survival that 11% of those with scores of nine or ten died within 6 months compared with at least 65% of those with scores of four or less. In terms of 12-month survival 54% of those with the highest scores died within this period compared with at least 78% of those with the lowest scores.

**Table I** Age, sex and site of cancer of patients notified to trial of co-ordinating care for terminally ill cancer patients with a life expectancy of under 1 year

| | *n (%)* |
|---|---|
| *Age group[a] (years)* | |
| 18–34 | 4 (2%) |
| 35–49 | 9 (4%) |
| 50–64 | 40 (17%) |
| 65–74 | 65 (28%) |
| ≥75 | 111 (48%) |
| *Sex* | |
| Male | 118 (51%) |
| Female | 112 (49%) |
| *Site of primary[b]* | |
| Lung | 57 (25%) |
| Colorectal | 41 (18%) |
| Breast | 32 (14%) |
| Prostate | 22 (10%) |
| Other | 76 (34%) |

[a]Unknown for one subject. [b]Unknown for two subjects.

**Figure 3** Survival curves for patients with differing Spitzer QL Index scores. —— QL 9, 10; --- QL 8; ····· QL 7; —— QL 6; --- QL 5; ····· QL 4; —— QLs 1,2,3.

**Table II** Six and 12-month survival rates[a] for patients with varying Spitzer QL Index scores at interview

| *Spitzer QL Index score* | *n* | *Six-month survival (95% confidence interval) %* | *Twelve-month survival (95% confidence interval) %* |
|---|---|---|---|
| 1, 2, 3 | 18 | 34 ( 9–58) | 17 (−10–44) |
| 4 | 33 | 35 (18–52) | 22 ( 6–38) |
| 5 | 44 | 65 (49–81) | 56 ( 40–72) |
| 6 | 41 | 53 (37–70) | 40 ( 23–57) |
| 7 | 42 | 56 (40–73) | 36 ( 18–55) |
| 8 | 35 | 76 (62–90) | 58 ( 40–76) |
| 9, 10 | 18 | 89 (74–104) | 46 ( 21–70) |

[a]Calculated using life table methodology.

*How well can the QL Index predict the likelihood of individual cancer patients dying within 6 months of assessment?*

All possible cut-off points on the QL Index were used to predict the likelihood of patients with scores above or below the cut-off dying within 6 months of interview. Table III gives the positive and negative predictive values together with the sensitivity and specificity rates associated with each cut-off.

Positive predictive values (that is the proportion of patients with scores at or below cut-off who died within 6 months) improved as the cut-off point on the QL Index decreased. In contrast negative predictive values (the proportion of patients with scores above cut-off who survived for longer than 6 months) declined as the cut-off point was lowered. For example, 73% of those with a QL Index score of three or less died within 6 months while 60% of those with a score of more than three lived for longer than this. In contrast 43% of those with scores of eight or less died within 6 months while 88% of those with scores of nine or ten lived for longer.

As the cut-off on the QL Index was raised the sensitivity of the test (the proportion of all patients dying within 6 months

**Table III** Accuracy of the Spitzer QL Index in predicting death within 6 months of interview: positive and negative predictive values and rates of sensitivity and specificity associated with using different cut-off points on the Index to distinguish patients likely to die within 6 months from those who will live longer

| *Cut-off point on Spitzer QL Index* | *Predictive values (%)* | | *Sensitivity (%)* | *Specificity (%)* |
|---|---|---|---|---|
| | *Positive* | *Negative* | | |
| 2 or less versus 3 or more | 75 | 60 | 4 | 99 |
| 3 or less versus 4 or more | 73 | 62 | 11 | 97 |
| 4 or less versus 5 or more | 64 | 65 | 31 | 88 |
| 5 or less versus 6 or more | 48 | 65 | 45 | 67 |
| 6 or less versus 7 or more | 48 | 70 | 67 | 51 |
| 7 or less versus 8 or more | 47 | 79 | 86 | 35 |
| 8 or less versus 9 or more | 43 | 88 | 97 | 14 |
| 9 or less versus 10 | 42 | 100 | 100 | 6 |

*n* = 181, total deaths in 6 months = 73. Patients were included in this analysis only if 6 months had elapsed between interview and the point of analysis (2 years after the beginning of the trial).

who had scores at or below cut-off) improved. The specificity (the proportion of all patients surviving 6 months who had scores above cut-off) was, however, reduced. A cut-off of three included 11% of all patients who died within 6 months while 97% of those who lived for longer had scores of more than three. In contrast a cut-off of eight included 97% of those who died within 6 months while 14% of those who lived for longer had scores greater than eight: 86% of those who did not die in 6 months were therefore inappropriately assigned to the 'dead in 6 months' group. Good sensitivity was therefore obtained at the cost of including a high proportion of patients who did not die within 6 months.

### Discussion

The first question addressed in this paper was how well doctors and nurses were able to distinguish between cancer patients with a life expectancy of more or less than 1 year. Results showed that errors were made in between 17 and 25% of cases. This suggests the need to search for other estimates of prognosis to complement clinical judgement.

Previous studies have shown that doctors and nurses tend to be overly optimistic when estimating life expectancy (Forster & Lynn, 1988; Parkes, 1972; Evans & McCarthy, 1985). Results of the present study suggest that staff are no more likely to over-estimate than to under-estimate life expectancy in cancer patients. The contrast between these results and those of other studies may be because the latter were concerned with estimating the precise length of life remaining to patients already predicted to have a limited prognosis, such as patients referred to a terminal care support team (Evans & McCarthy, 1985). In contrast, patients in the present study were drawn from the general population of cancer patients in contact with a district general hospital.

Although estimates of life expectancy in this study were inaccurate in approximately 23% of all patients, the proportion who were given a prognosis of less than 1 year corresponded closely to the proportion who died within a year (41% versus 38%). This has important implications for the planning of services for the terminally ill. If Wandsworth Health Authority had used the judgements of prognosis made by the medical and nursing staff to estimate the need for such services they would have found the demand for these services to be very close to the estimate. Such estimates may, therefore, be sufficiently accurate to be of use to those planning services despite the fact that they are inaccurate for approximately one in five individual cancer patients.

The second question was whether medical and nursing staff differed in their ability to estimate the prognosis of cancer patients. The results showed that there were no significant differences in the length of survival of patients given a prognosis of over 1 year by doctors and that of those given a similar prognosis by nurses. The same was true for patients given a prognosis of under 1 year. The doctors and nurses who took part in this study did not, therefore, appear to differ in their ability to estimate the prognosis of patients.

However, it should be noted that doctors mainly estimated the prognosis of patients attending outpatient clinics, while most of prognostic estimates made by nursing staff were for inpatients. The information available to staff during a brief contact with a patient in an outpatient clinic may differ in both kind and depth to that available to staff who care for a patient during an inpatient stay. The medical and nursing staff in this study may therefore have had different types and amounts of information available to them. In addition, the outpatients were likely to be less severely ill at the time of assessment than inpatients. This means that the medical and nursing staff did not make estimates of prognosis for truly comparable groups of patients. Evidence for this comes from the fact that, although 12-month survival rates were very similar in the two groups, more patients given a prognosis of under 1 year by nursing staff than by medical staff died within 6 months, indicating that this group contained more severely ill patients. Caution is therefore needed in interpreting these results: differences in the ability of medical and nursing staff to make estimates of prognosis may have emerged if these estimates had been made for comparable groups of patients and if the same sources of information had been available to both doctors and nurses.

The third question was whether there were differences in the length of life remaining when groups of patients with different QL Index scores were compared. Although too much emphasis should not be placed on the results because of large confidence intervals, results suggest that patients with low QL Index scores were more likely to die within 6 months of interview than those with high scores. This is consistent with the results of the National Hospice Study (Morris *et al.*, 1986; Morris & Sherwood, 1987) which found that groups of patients close to death had lower mean QL Index scores than those who survived longer. These results suggest that the QL Index may be useful in reducing prognostic uncertainty in terminal care. This possibility was explored by investigating whether scores on the QL Index could be used to predict the likelihood of cancer patients dying within 6 months of assessment. It was found that the QL Index was not a strong predictor of this in individual patients. It may concluded that by itself the QL Index is not sufficiently accurate to be used when making important decisions about treatment and care. It might, however, make a valuable contribution to the decision-making process when combined with other information already available to clinicians.

Evidence from previous studies suggest that knowledge of the patient's sex, age, primary tumour type or site of metastases may contribute little to the prediction of survival times (Reuben *et al.*, 1988) and that the same is true for psychological factors such as feelings of hopelessness, life satisfaction and the amount of adjustment needed in response to initial diagnosis (Cassileth *et al.*, 1988). Instead, it has been suggested that clinical features such as performance status and the presence of certain symptoms may give a better idea of prognosis (Cassileth *et al.*, 1988; Reuben *et al.*, 1988). For instance, Reuben *et al.* (1988) found that the predictive power of the Karnofsky Index was improved when combined with five symptoms (shortness of breath, problems eating or loss of appetite, trouble swallowing, dry mouth and weight loss) which had independent predictive value. They suggest that these symptoms are indicative of a 'terminal cancer syndrome' which spans across tumour type and metastases sites. The predictive value of the QL Index may therefore similarly be improved by the addition of symptoms such as these.

Those who wish to use the QL Index as a predictor of life expectancy will need to decide which cut-off point on the QL Index is most useful. The decision will depend upon the implications of mis-classification. It might be preferable to treat patients who live longer than 6 months as if they were going to die within this period than to risk doing the reverse. Such patients might benefit if, for instance, they are referred to a hospice or home care team earlier and therefore have a longer period to develop good communications with the staff (Evans & McCarthy, 1984). In this case a cut-off point which provided good sensitivity would be selected in order to reduce the likelihood of appropriate terminal care being provided too late. Alternatively, it may be decided that it is undesirable to erroneously label a patient as likely to die within 6 months who will in fact live for longer. In this case it would be advisable to adopt a cut-off point on the QL Index which has high specificity. Whether the QL Index is felt to have value in reducing prognostic uncertainty in terminal care and, if so, which cut-off should be adopted, will therefore depend partly on whether it is considered better to include everyone with any likelihood of dying within 6 months or to include only those who are almost certain to die within this period.

This issue is of more than theoretical interest and has important implications in the light of current trends in health care provision. In the USA if patients are classified as having a life expectancy of less than 6 months but then live longer than this the cost of their hospice care may not continue to be re-imbursed by Medicare. Such patients could prove expensive to hospice programmes. If patients who die within 6 months are wrongly classified as being likely to live longer than this they may be denied entry to a hospice programme which may have been beneficial. Pearlman (1988) has discussed this dilemma and argues that those concerned with terminal care should determine acceptable error rates before deciding how to assess prognosis.

Accurate prognostic information is important to those planning and providing services as well as to clinicians concerned with individual patients. It has already been noted that the proportion of patients estimated by medical and nursing staff as being likely to die within a year is very close to the proportion who actually die in this period. The addition of information from the QL Index, such as the proportion of these patients who have very low scores and are therefore likely to die in the near future, may further improve the ability to predict group survival rates. This information would be of use to those planning services who require information on levels of need in a population.

The QL Index is already regarded as a valuable summary measure of quality of life in cancer patients (Clark & Fallowfield, 1986). The results presented here suggest that it may also be of some use in reducing prognostic uncertainty in terminal care.

This study was supported by a grant from the Medical Research Council. We acknowledge with thanks the help of our research interviewers J. Ochera, E. Rink and J. Taylor.

### References

BARNETT, M. & MCCARTHY, M. (1987). Identification of terminally ill patients in the community. In *1986 International Symposium on Pain Control*, Doyle, D. (ed.) p. 77. Royal Society of Medicine: London.

CASSILETH, B.R., WALSH, W.P. & LUSK, E.J. (1988). Psychosocial correlates of cancer survival: a subsequent report 3 to 8 years after cancer diagnosis. *J. Clin. Oncol.*, **6**, 1723.

CLARK, A. & FALLOWFIELD, L.F. (1986). Quality of life measurements in patients with malignant disease. *J. R. Soc. Med.*, **79**, 165.

EVANS, C. & MCCARTHY, M. (1984). Referral and survival of patients accepted by a terminal care support team. *J. Epidemiol. Comm. Health*, **38**, 310.

EVANS, C. & MCCARTHY, M. (1985). Prognostic uncertainty in terminal care: can the Karnofsky Index help? *Lancet*, **i**, 1204.

FORSTER, L.E. & LYNN, J. (1988). Predicting life span for applicants to inpatient hospices. *Arch. Intern. Med.*, **148**, 2540.

LUNT, B. & HILLIER, R. (1981). Terminal care: present services and future priorities. *Br. Med. J.*, **283**, 595.

MACDONALD, L.D. (1989). A new scheme for the care of terminally ill cancer patients. *MRC News*, **44**, 12.

MOR, V. (1987). Cancer patients' quality of life over the disease course: lessons from the real world. *J. Chronic Dis.*, **40**, 535.

MOR, V., LALIBERTE, L., MORRIS, J.N. & WEIMAN, M. (1984). The Karnofsky performance status scale: an examination of its reliability and validity in a research setting. *Cancer*, **53**, 2002.

MORRIS, J.N. & SHERWOOD, S. (1987). Quality of life of cancer patients at different stage in the disease trajectory. *J. Chronic Dis.*, **40**, 545.

MORRIS, J.N., SUISSA, S., SHERWOOD, S., WRIGHT, S.M. & GREER, D. (1986). Last days: a study of the quality of life of terminally ill cancer patients. *J. Chronic Dis.*, **39**, 47.

PADILLA, G.V., PRESENT, C., GRANT, M.M., METTER, G., LIPSETT, J. & HEIDE, F. (1983). Quality of life index for patients with cancer. *Res. Nurs. Health*, **6**, 117.

PARKES, C.M. (1972). Accuracy of predictions of survival in later stages of cancer. *Br. Med. J.*, **ii**, 29.

PEARLMAN, R.A. (1988). Inaccurate predictions of life expectancy. Dilemmas and opportunities (editorial). *Arch. Intern. Med.*, **148**, 2537.

PETO, R., PIKE, M.C., ARMITAGE, P. & 7 others (1977). Design and analysis of randomised clinical trials requiring prolonged observations of each patient. *Br. J. Cancer*, **35**, 1.

REUBEN, D.B., MOR, V. & HIRIS, J. (1988). Clinical symptoms and length of survival on patients with terminal cancer. *Arch. Intern. Med.*, **148**, 1586.

SLEVIN, M.L., PLANT, H., LYNCH, D., DRINKWATER, J. & GREGORY, W.M. (1988). Who should measure quality of life, the doctor or the patient? *Br. J. Cancer*, **57**, 109.

SPITZER, W.O., DOBSON, A.L., HALL, J. & 5 others (1981). Measuring the quality of life of cancer patients: a concise QL-Index for use by physicians. *J. Chronic Dis.*, **34**, 585.

YATES, J.W., CHALMER, B. & MCKEGNEY, F.P. (1980). Evaluation of patients with advanced cancer using the Karnofsky performance status scale. *Cancer*, **45**, 2220.